# Flow Cytometry-Assessed PD1/PDL1 Status in Tumor-Infiltrating Lymphocytes: A Link With the Prognosis of Diffuse Large B-Cell Lymphoma

**DOI:** 10.3389/fonc.2021.687911

**Published:** 2021-06-15

**Authors:** Zihang Chen, Xueqin Deng, Yunxia Ye, Wenyan Zhang, Weiping Liu, Sha Zhao

**Affiliations:** Department of Pathology, West China Hospital, Sichuan University, Chengdu, China

**Keywords:** diffuse large B-cell lymphoma, PD1, PDL1, tumor-infiltrating lymphocytes, flow cytometry

## Abstract

The PD1/PDL1 status of tumor-infiltrating lymphocytes (TILs) in diffuse large B-cell lymphoma (DLBCL) reflects immune function. However, the previously reported methods for evaluating this status are complex and may not be widely used in clinical practice. In addition, these studies did not introduce healthy controls to designate the cut-off when evaluating the prognostic value of the status. In this study, we retrospectively evaluated the PD1/PDL1 status in TILs of 24 DLBCL tissue samples and normal immune cells in 61 demographically matched healthy controls (tissue samples from patients with reactive hyperplasia [RH]) by flow cytometry. We investigated the prognostic value of the PD1/PDL1 status in TILs by precisely determining the cut-off value and assessing the reliability of flow cytometry. The mean fluorescence intensity (MFI) of PD1 in TIL-T-cells (TIL-Ts; median, 110) and CD8+TIL-Ts (median, 64) was significantly higher than that of CD3+T-cells (median, 64) and CD8+ T-cells (median, 34) in RH. The cut-off values of PD1/PDL1 status for analyzing prognostic values were defined considering the PD1/PDL1 status of samples from both patients with DLBCL and healthy controls. High MFI of PD1 in TIL-Ts (MFI >108, P = 0.022), high proportion of PD1+CD4+TIL-Ts (>1.1% of CD4+TIL-Ts, P = 0.049), high proportion of PD1+CD8+TIL-Ts (>2% of CD8+TIL-Ts, P = 0.025), and high MFI of PDL1 in TIL-Ts (MFI >83, P = 0.023) were risk factors for inferior prognosis of DLBCL. Our results indicate that flow cytometry is a reliable and convenient method for evaluating the immune-checkpoint status of TILs, which probably holds major implications in clinical practice.

## Introduction

The composition and proportion of tumor-infiltrating lymphocytes (TILs) reflect the immune function of the tumor microenvironment (TME) (1). The correlation between TILs and overall survival (OS) has been demonstrated in many solid tumors and hematopoietic malignancies (2, 3). However, the prognostic value of TILs differs across tumor types, even in cohorts of the same tumor (4–6). It also has been recognized that TILs participate in the development, progression, and prognosis of diffuse large B-cell lymphoma (DLBCL) (7). In a previous study on *de novo* DLBCL, we found that patients with high tumor-infiltrating T-cells (TIL-Ts), especially high CD8+TIL-Ts, had prolonged OS (2). However, recent studies have reported that the expression of PD1 in TIL-Ts of lymphoma possibly counteracts this positive effect on OS by dampening immune surveillance (3). Additionally, PDL1, which was previously thought to be expressed on tumor cells and macrophages, has been proved to be expressed on TILs in many tumor types, including bladder carcinoma, head and neck carcinoma, breast carcinoma, and DLBCL (4, 8). Therefore, determining the PD1/PDL1 status of TILs can prove helpful when assessing the risk of patients and developing therapeutic strategies for DLBCL. However, methods used in previous studies for evaluating PD1/PDL1 status, such as multiplex fluorescent or multiplex immunohistochemistry, are complex [4], and may not be widely used in clinical practice. In addition, these reports did not introduce the baseline PD1/PDL1 status of healthy controls when making the cut-off value. Flow cytometry, which exhibits high consistency, good repeatability, and convenient operation, is especially suitable for assessing the PD1/PDL1 status of TILs. In this study, we sought to determine the mean fluorescence intensity (MFI) and categorize proportion of PD1/PDL1-positive TILs in a small cohort of patients with DLBCL using flow cytometry. In addition, we aimed to compare the results to those of healthy controls to define the cut-off value when analyzing the prognostic value of the PD1/PDL1 status.

## Methods

The study included tissue samples from 24 patients with histologically confirmed DLBCL and 61 patients with reactive hyperplasia (RH) from January 2017 to December 2018 at West China Hospital, Sichuan University. Samples from patients with RH with matched demographic baseline characteristics to those of DLBCL were considered as healthy controls (**Supplementary Material, Table S1**). Relevant clinical information was obtained from electronic medical records, and follow-up information was collected from telephone interviews and/or electronic medical records in March 2020. Survival time was calculated from the date of diagnosis to the date of death or the last follow-up. All biopsied specimens were subjected to flow cytometry. The methods for sample preparation, staining and acquisition, data analysis, and identification of TILs have been previously described (9, 10). Flow cytometry data were obtained using BD FACSCanto II equipped with two lasers (blue and red), and analyzed with FACSDiva software. The fluorescent-labeled antibodies were obtained from BD, and detailed information regarding the antibodies is listed in **Table S2** (**Supplementary Material**).

Statistical analysis was performed using SPSS software (version 24.0; SPSS Corp., Chicago, IL, USA). Continuous variables were analyzed using the Mann–Whitney test or independent sample t-test depending upon the data distribution type; categorical variables were compared using Fisher’s exact test. The cut-off scores for analyzing prognostic values were defined considering the PD1/PDL1 status of both patients with DLBCL and healthy controls (Supplementary Material). Survival data were calculated using the Kaplan–Meier estimator. Log-rank test was used to compare different survival functions; Cox’s proportional hazard regression model to estimate the hazard rates of the prognostic factors. Tests were considered significant at two-sided probability values less than 0.05 (P <0.05).

## Results

The baseline information of the patients with DLBCL is shown in **Table 1**. The median age was 55 years (range, 19–80 years), and the male to female ratio was 1.4:1. Non-germinal center B-cell (non-GCB) type was identified in 17 samples (71%), whereas early stage (stage I/II) in 14 cases (64%). Fifteen cases (68%) presented extranodal lesion, and only one case (5%) was categorized “very good” according to the revised International Prognostic Index. All patients underwent R-CHOP or R-CHOP-like chemotherapy.

**Table 1:** The baseline characteristics of patients with diffuse large B-cell lymphoma (DLBCL).

	All patients n (%)	MFI of CD3+PD1+T-cells , n (%)			Percentage of CD4+PD1+T-cells , n (%)			Percentage of CD8+PD1+T-cells , n (%)			MFI of CD3+PDL1+T-cells , n (%)		
		High	Low	P	High	Low	P	High	Low	p	High	Low	P
**Age(y), median (range)**	55 (19–80)	60 (36–80)	49 (19–75)	0.290	56 (45–80)	45 (19–75)	0.295	45 (19–80)	54 (36–75)	0.689	53 (19–80)	57 (35–79)	0.452
**Sex (M/F)**	14/10	10/8	4/5	0.285	3/0	4/8	0.128	3/3	4/5	1.000	9/7	5/3	1.000
**Hans**				***0.028***			0.333			1.000			0.352
**GCB**	7 (29)	2 (13)	5 (56)		0 (0)	3 (25)		1 (17)	2 (22)		6 (37)	1 (12)	
**Non-GCB**	17 (71)	13 (87)	4 (44)		3 (100)	9 (75)		5 (83)	7 (78)		10 (63)	7 (88)	
**R-IPI**				0.524			0.188			0.709			0.348
**Very Good**	1 (5)	1 (7)	0 (0)		0 (0)	1 (10)		0 (0)	1 (13)		0 (0)	1 (14)	
**Good**	13 (62)	10 (67)	3 (50)		3 (100)	4 (40)		3 (60)	4 (50)		9 (64)	4 (57)	
**Poor**	7 (33)	4 (27)	3 (30)		0 (0)	5 (50)		2 (40)	3 (38)		5 (36)	2 (29)	
**Stage**				0.665			0.923			0.266			0.649
**I/II**	14 (64)	10 (67)	4 (57)		2 (67)	7 (64)		2 (40)	7 (78)		8 (57)	6 (75)	
**III/IV**	8 (36)	5 (33)	3 (43)		1 (33)	4 (36)		3 (60)	2 (22)		6 (43)	2 (25)	
**Extranodal lesion**				0.823			0.923			0.580			1.000
**None**	7 (32)	5 (33)	2 (29)		1 (33)	4 (36)		1 (20)	4 (44)		4 (29)	5 (63)	
**Present**	15 (68)	10 (67)	5 (71)		2 (67)	7 (78)		4 (80)	5 (56)		10 (71)	3 (38)	
**B symptoms**													
**Fever**	5 (23)	1 (7)	4 (57)	***0.009***	0 (0)	1 (9)	1.000	0 (0)	1 (11)	1.000	4 (29)	1 (13)	0.613
**Night sweet**	0 (0)	0 (0)	0 (0)	1.000	0 (0)	0 (0)	1.000	0 (0)	0 (0)	1.000	0 (0)	0 (0)	1.000
**Lose weight**	2 (9)	1 (7)	1 (14)	0.563	0 (0)	1 (9)	1.000	0 (0)	1 (11)	1.000	1 (7)	1 (13)	1.000
**Splenomegaly**	4 (18)	2 (13)	2 (29)	0.388	0 (0)	1 (9)	1.000	0 (0)	1 (11)	1.000	3 (21)	1 (13)	1.000
**Hepatomegaly**	0 (0)	0 (0)	0 (0)	1.000	0 (0)	0 (0)	1.000	0 (0)	0 (0)	1.000	0 (0)	0 (0)	1.000
**BM involvement**	1 (5)	0 (0)	1 (20)	0.086	0 (0)	0 (0)	1.000	0 (0)	0 (0)	1.000	1 (8)	0 (0)	1.000
**Lab test, median (range)**													
**WBC (×10^9^/L)**	5.4 (1.6–42.6)	5.4 (1.62–8.79)	5.4 (2.9–42.6)	0.347	5.3 (1.6–7.4)	5.4 (2.4–16.4)	0.448	4.5 (1.6–7.6)	5.4 (2.9–16.4)	0.181	5.4 (1.6–42.6)	6.3 (2.9–16.4)	0.417
**RBC (×10^9^/L)**	3.9 (2.1–5.3)	4.3 (2.1–5.3)	3.7 (3.2–4.4)	0.318	4.6 (3.2–5.3)	3.8 (2.4–5.3)	0.365	3.7 (2.4–5.3)	3.8 (3.2–5.3)	0.607	3.9 (2.2–5.3)	4.1 (3.2–5.3)	0.452
**PLT (×10^9^/L)**	144 (5–802)	137 (5–259)	153 (5–802)	0.726	209 (56–226)	145 (5–802)	0.633	217 (26–802)	137 (5–215)	0.181	144 (26–802)	140 (5–259)	0.697
**AST (IU/L)**	23 (13–134)	19 (13–134)	29.5 (20–94)	***0.023***	16 (15–25)	25 (13–134)	0.371	16 (13–134)	25 (14–58)	0.435	22 (13–134)	24 (14–58)	0.799
**ALT (IU/L)**	18 (5–161)	15 (5–161)	29 (18–36)	0.095	11 (11–17)	29 (5–161)	0.112	11 (5–161)	29 (13–86)	0.171	16 (5–161)	25 (9–41)	0.636
**TB (μmol/L)**	10.7 (5.3–67.3)	10 (5.3–67.3)	17 (5.6–30.8)	0.470	11 (5.3–11.6)	8.5 (6.6–20.6)	0.937	8 (5.3–11.6)	10 (6.6–20.6)	0.354	10.4 (5.3–67.3)	17.9 (6.1–24.8)	0.636
**ALP (IU/L)**	71 (35–334)	70 (35–334)	77.5 (54–118)	0.622	64 (54–71)	66 (35–255)	0.811	64 (35–255)	66 (40–95)	0.833	74 (35–334)	57 (40–86)	0.322
**LDH (IU/L)**	228 (105–2,263)	209 (105–1,369)	302 (148–2,263)	0.267	319 (153–490)	219 (148–1,369)	1.000	362 (153–1,369)	192 (148–483)	0.171	298 (153–2,263)	199 (105–483)	0.128

Bold value indicates statistically significant (P < 0.05).

**Figure 1:**
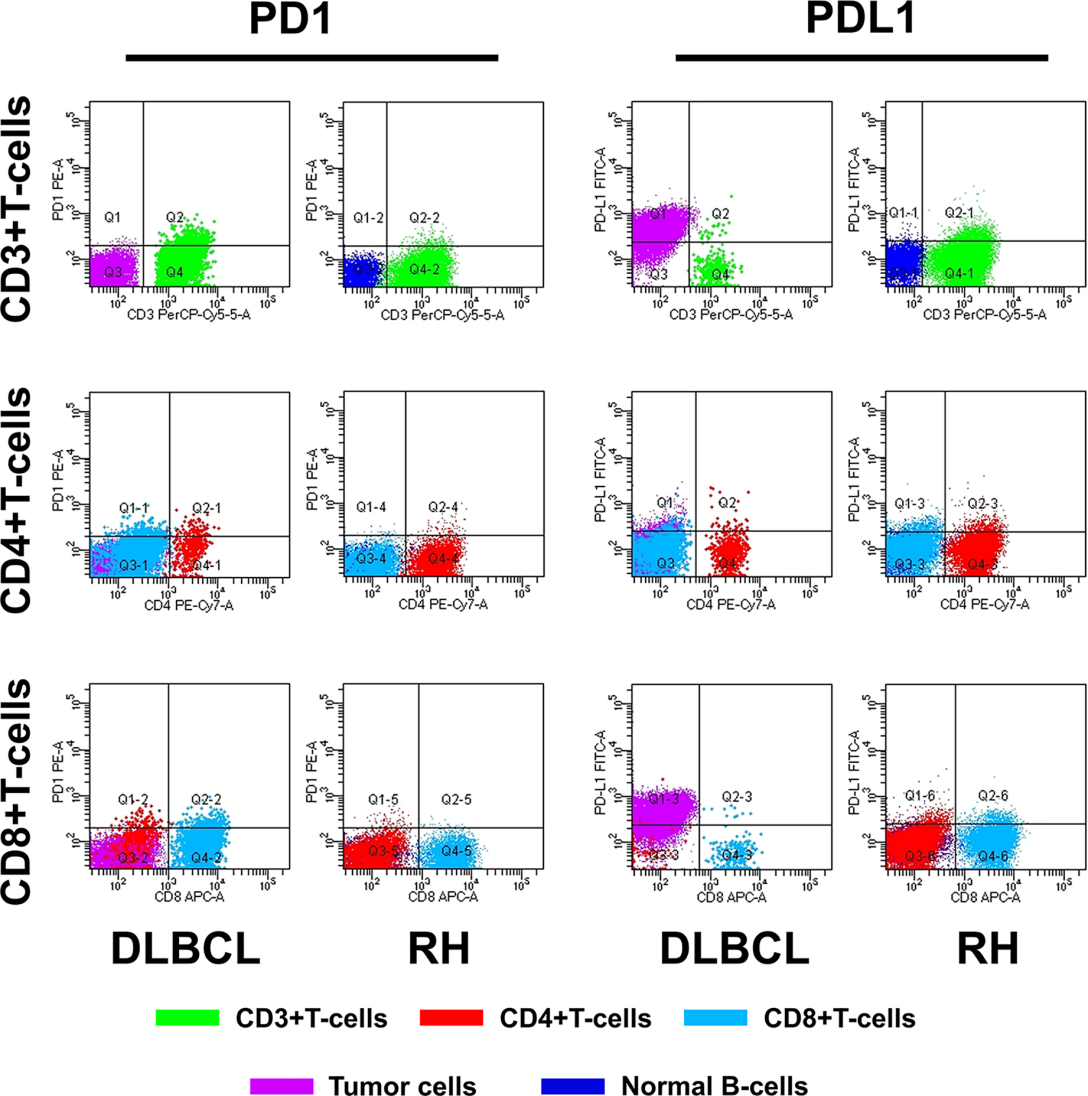
Flow cytometry analysis of PD1/PDL1 status in TILs and normal immune cells.

**Figure 2:**
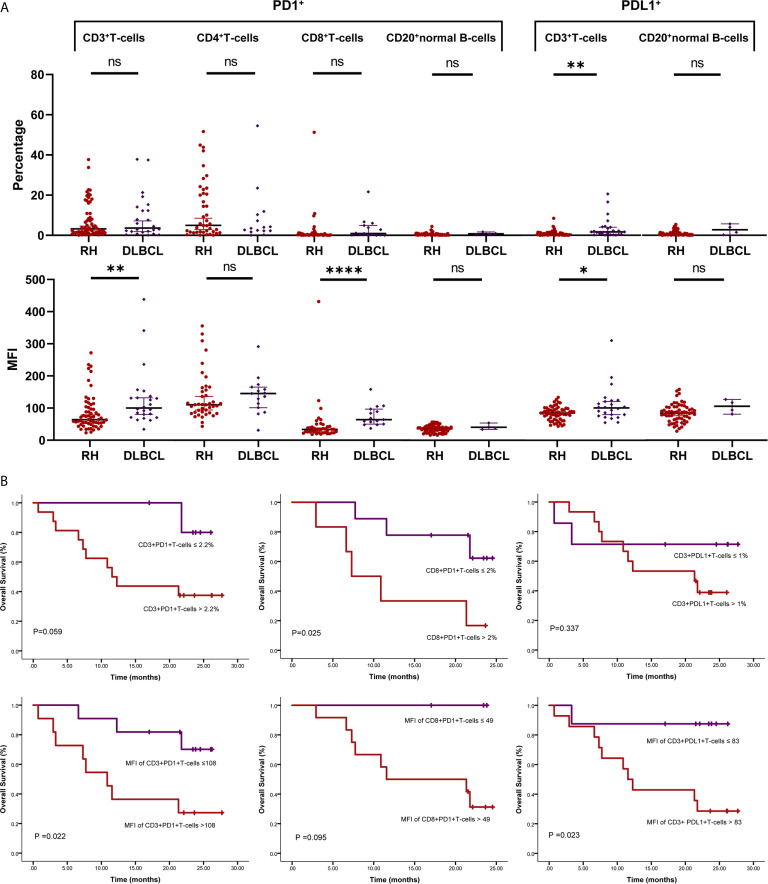
PD1/PDL1 status in tumor-infiltrating lymphocytes (TILs) in diffuse large B-cell lymphoma (DLBCL). **(A)** Scatter plots depicting PD1/PDL1 status in reactive hyperplasia (RH) and diffuse large B-cell lymphoma (DLBCL). *P < 0.05; **P < 0.005; ****P < 0.0001. **(B)** Prognostic value of PD1/PDL1 status of TILs in DLBCL. ns, no significant.

The method of evaluating the PD1/PDL1 status of each TIL and its counterpart cell in RH is shown in **Figure 1**. Regarding PD1 status, no significant difference was observed among the proportions of PD1+TIL-Ts (PD1+TIL-Ts/TIL-Ts), PD1+ CD4+TIL-Ts (PD1+CD4+TIL-Ts/CD4+TIL-Ts), PD1+ CD8+TIL-Ts (PD1+CD8+TIL-Ts/CD8+TIL-Ts), and PD1+TIL-B-cells (TIL-Bs, PD1+TIL-Bs/TIL-Bs) in DLBCL cells and their counterpart cells in RH. Nevertheless, the MFI of PD1 in TIL-Ts (median, 110) and CD8+TIL-Ts (median, 64) was significantly higher than that of CD3+T-cells (median, 64) and CD8+T-cells (median, 34) in RH (**Table 2** and **Figure 2A**).

**Table 2:** Univariate and multivariate survival analyses of PD1/PDL1 expression in tumor-infiltrating lymphocytes (TILs) of diffuse large B-cell lymphoma (DLBCL) evaluated by flow cytometry within each cell type.

		Median Value		Risk Factor, cut-off definition	Univariate Analysis	Multivariate Analysis	
		RH	DLBCL			HR (95% CI)	*P*
**PD1 expression**							
**T-cells**	%	3.3	3.8	High PD1+TIL-Ts >2.2% of TIL-Ts	Poorer OS, P = 0.059	–	–
	MFI	64	110	High MFI of PD1 in TIL-Ts MFI >108	Poorer OS, P = 0.022	1.58 (0.32–7.46)	0.581
**CD4+T-cells**	%	5.7	3.5	High PD1+CD4+TIL-Ts >1.1% of CD4+TIL-Ts	Poorer OS, P = 0.049	1.69 (0.26–10.87)	0.579
	MFI	110	145	High MFI of PD1 in CD4+TIL-Ts MFI >150	Poorer OS, P = 0.128	–	–
**CD8+T-cells**	%	0.2	0.9	High PD1+CD8+TIL-Ts >2% of CD8+TIL-Ts	Poorer OS, P = 0.025	3.50 (0.57–21.28)	0.286
	MFI	34	64	High MFI of PD1 in CD8+TIL-Ts MFI >49	Poorer OS, P = 0.095	–	–
**Normal B-cells**	%	0.3	0.8	–	–	–	–
	MFI	36	41	–	–	–	–
**PDL1 expression**							
**T-cells**	%	0.5	1.7	High PDL1+TIL-Ts >1% of TIL-Ts	Poorer OS, P = 0.337	–	–
	MFI	81	101	High MFI of PDL1 in TIL-Ts MFI >83	Poorer OS, P = 0.023	6.67 (0.81–55.56)	0.078
**Normal B-cells**	%	0.5	2.8	–	–	–	–
	MFI	84	106	–	–	–	–

For PDL1 status, the proportion of PDL1+TIL-Ts (PDL1+TIL-Ts/TIL-Ts; median, 1.7%) and MFI of PDL1 in TIL-Ts (median, 101) in DLBCL were significantly higher than those of the counterpart cells in RH. Similar trends were observed on comparing TIL-Bs (MFI and proportion: PDL1+TIL-Bs/TIL-Bs) in DLBCL and normal B-cells in RH (**Table 2** and **Figure 2A**).

The cut-off scores and prognostic values of each risk factor are shown in **Table 2** and **Figure 2B**. Univariate analysis revealed that among the measured candidate risk factors, high MFI of PD1 in TIL-Ts (MFI >108, P = 0.022), high proportion of PD1+CD4+TIL-Ts (>1.1% of CD4+TIL-Ts, P = 0.049), high proportion of PD1+CD8+TIL-Ts (>2% of CD8+TIL-Ts, P = 0.025), and high MFI of PDL1 in TIL-Ts (MFI >83, P = 0.023) were risk factors for inferior prognosis of DLBCL. A high proportion of PD1+TIL-Ts (>2.2% of TIL-Ts) and high MFI of PD1 in CD8+TIL-Ts (MFI >49) were marginally associated with a shorter OS (P = 0.059 and 0.095, respectively). All factors selected by univariate analysis were included in the multivariate analysis. However, no independent risk factors were found to be associated with OS.

Patients with high MFI of PD1 in TIL-Ts showed a significantly higher prevalence of non-GCB type (P = 0.028) and a lower rate of presenting fever (P = 0.009) than those with low MFI. No significant differences in baseline levels were detected between the other pairs of corresponding groups (**Table 1**).

## Discussion

Evaluating the immune-checkpoint status of the TME is urgently needed in cancer clinical practice (11). Previously, *in situ* detection using multiplex immunofluorescent or multiplex immunohistochemistry methods to simultaneously quantify immune-checkpoint molecules on individual cells within the TME has been reported. These methods have shown both high consistency and repeatability to indicate the OS; however, they involve complex and time-consuming processes such as multiround staining, image acquisition to dye inactivation, and digital image analysis (4, 8). Flow cytometry not only overcomes these limitations, but is also convenient (12). To the best of our knowledge, the current study is the first to evaluate the PD1/PDL1 status of TILs in tumor and normal tissue samples using flow cytometry. Our results are consistent with those of the abovementioned studies, suggesting that flow cytometry is indeed a reliable method for evaluating the immune-checkpoint status of TILs, which can be extensively used in clinical practice in the future.

In the present study, the baseline level of PD1/PDL1 status in healthy controls corresponded to normal immune function, thereby increasing the evidence of tumorigenesis. In addition, the baseline level can help to precisely define immune function of the TME and may facilitate defining the cut-off value when implementing PD1/PDL1 blockade immunotherapy. However, previous studies did not consider the corresponding normal tissue when investigating the TME of DLBCL cells. Therefore, we introduced RH tissue samples as “healthy” controls to evaluate PD1/PDL1 status in normal immune cells. Similar to the investigations based on the peripheral blood of healthy donors, all detected types of host immune cells express PD1 and PDL1 to various extents, whereas the main type of immune cell expressing PD1/PDL1 is T-cell (13). Compared with DLBCL tissue samples, the differences were mainly in the MFI of PD1/PDL1 rather than the proportion of PD1/PDL1+ host immune cells. Therefore, it is speculated that tumor cells may upregulate PD1/PDL1 expression in individual TILs rather than recruit more PD1/PDL1-expressing immune cells to enhance tumorigenesis.

The current study indicated that an increased proportion of PD1/PDL1 + TIL-Ts and high MFI of PD1/PDL1 in TIL-Ts is linked to adverse prognostic effects in patients with DLBCL. Corroborating our results, dysfunction of immune surveillance by PD1/PDL1 expression in TILs has been previously reported indispensable for tumor survival (8).

Although our study has several strengths, there are a few limitations associated as well. One major shortcoming is the small cohort size, which was dictated by sample availability. Since fresh tissues rather than paraffin-embedded samples are required for flow cytometry analysis, a retrospective study with a large number of tissue samples could not be performed. Additionally, we did not evaluate the subsets of TIL-Ts in detail. Some host T-cells in the TME, especially helper T-cells, naturally express PD1, which could influence the results.

In conclusion, we could evaluate the PD1/PDL1 status in the TME of DLBCL using a clinically accessible method. This study provides valuable insights for further flow cytometry-based investigations of the immune function of TILs in DLBCL. Future research should validate these findings in larger sized cohorts to measure the risk of patients and thereby guide immunotherapy by comprehensively evaluating the immune status of the TME in DLBCL.

## Data Availability Statement

The datasets presented in this article are not readily available. Requests to access the datasets should be directed to: (hxblzhaosha@126.com).

## Ethics Statement

The study was approved by the Ethics Committee on Biomedical Research, West China Hospital of Sichuan University. The committee waived the requirement for informed consent as the data for the patients included in the study were retrospectively analyzed. 

## Author Contributions

ZC: Conceptualization, Formal analysis, Writing—Original Draft, Visualization, Funding acquisition; XD: Investigation, Data Curation, Validation; YY, WZ and WL: Resources, Writing—Review and Editing; SZ: Conceptualization, Supervision, Writing—Reviewing and Editing, Funding acquisition. All authors contributed to the article and approved the submitted version.

## Funding

This work was supported by the National Natural Science Foundation of China (30900534 to SZ, 81900195 to ZC), Sichuan Science and Technology Program (2018JY0612 to SZ), 1·3·5 project for disciplines of excellence—Clinical Research Incubation Project, West China Hospital, Sichuan University (2021HXFH027 to SZ), and Post-Doctor Research Project, West China Hospital, Sichuan University (2019HXBH067 to ZC).

## Conflict of Interest

The authors declare that the research was conducted in the absence of any commercial or financial relationships that could be construed as a potential conflict of interest.

## Supplementary Material

Supplementary Table 1 Demographic features matched between patients with reactive hyperplasia (RH) and those with diffuse large B-cell lymphoma (DLBCL).

## Abbreviations

DLBCL, diffuse large B-cell lymphoma; MFI, mean fluorescence intensity; Non-GCB, non-geminal center B-cell; OS, overall survival; RH, reactive hyperplasia; TILs, tumor-infiltrating lymphocyte; TIL-Ts, tumor-infiltrating T-cells; TIL-Bs, tumor-infiltrating B-cells; TME, tumor microenvironment.
