# Supplementary material for: Flow Cytometry-Assessed PD1/PDL1 Status in Tumor-Infiltrating Lymphocytes: A Link With the Prognosis of Diffuse Large B-Cell Lymphoma
Source: Front Oncol. 2021 Jun 15;11:687911. doi: 10.3389/fonc.2021.687911 (PMC8239303; doi:10.3389/fonc.2021.687911)
Supplement: Supplementary Table 1 — Demographic features matched between patients with reactive hyperplasia (RH) and those with diffuse large B-cell lymphoma (DLBCL). [file DataSheet_1.pdf]

Flow cytometry-assessed PD1/PDL1 status in tumor-infiltrating lymphocytes: A link with the prognosis of diffuse large B-cell lymphoma

Zihang Chen, M.D.[1#]; Xueqin Deng, M.D.[1#]; Yunxia Ye, M.D.[1]; Wenyan Zhang, Ph.D.[1], Weiping Liu, M.D.[1], Sha Zhao, Ph.D.[1]

1.Department of Pathology, West China Hospital, Sichuan University, No. 37 GuoXue Xiang, Chengdu, Sichuan 610041, China.

Supplementary methods

Table s1: Demographic features were matched between RH and DLBCL cases

	DLBCL (n=24)	RH (n=61)	*P*
**Age (year) median (range)**	55 (19-80)	48 (16-77)	0.576
**Sex**			0.171
**Male**	14	39	
**Female**	10	22	

Table s2: Antibody reagents for this study

Antibody	clonal	Fluorescence	Manufacturer
CD3	SK7	Percp-cy5-5	BD
CD4	SK3	PE-cy7	BD
CD8	SK1	APC	BD
CD19	SJ25C1	Percp-cy5-5	BD
CD20	L27	APC-H7	BD
CD45	2D1	APC-cy7	BD
Kappa	TB28-2	FITC	BD
Lambda	1-155-2	PE	BD
PD1 (CD279)	MIH4	PE	BD
PDL1 (CD274)	MIH1	FITC	BD

Setting up the cut-off

The methodology of setting up the cut-off was based on previous studies[1-3]. First, the performance of each feature (proportion and MFI of each kind of PD1+ or PDL1+ TILs) in predicting OS was evaluated through generating receiver operating characteristic (ROC) curves using simple logistic regression models. Then, the point where the sensitivity plus specificity was maximum in the ROC curves for predicting OS was calculated (optimal point). For each feature, if there was no significant difference between RH and DLBCL samples (PD1+TIL-Ts proportion, PD1+CD4+TIL-Ts proportion, PD1+CD8+TIL-Ts proportion, and MFI of PD1 in PD1+CD4+TIL-Ts), the optimal point was used as the cut-off. For the other features for which a significant difference was detected between RH and DLBCL samples (MFI of PD1 in PD1+TIL-Ts and PD1+CD8+TIL-Ts, MFI of PDL1 in PDL1+TIL-Ts), if the optimal point was in the range between the median value of the feature in RH and DLBCL, the optimal point was used as the cut-off. If not, the cut-off was defined as the point in the range between the median value of the feature in RH and DLBCL, where the sensitivity plus specificity was maximum in the ROC curves for predicting OS. The flowchart briefly illustrates the method of setting up the cut-off.

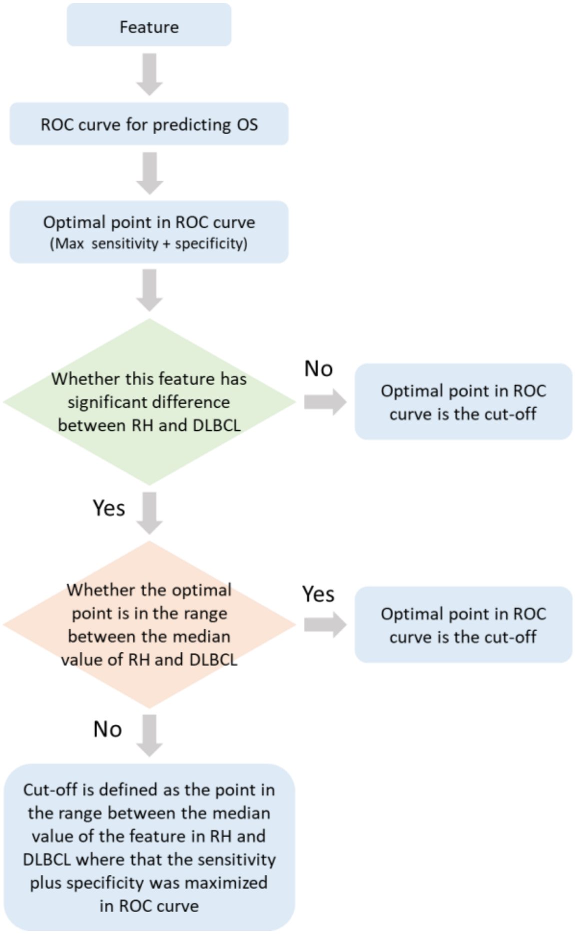

Reference

[1] Gomez-Gelvez JC, Salama ME, Perkins SL, Leavitt M, Inamdar KV. Prognostic Impact of Tumor Microenvironment in Diffuse Large B-Cell Lymphoma Uniformly Treated With R-CHOP Chemotherapy. Am J Clin Pathol. 2016;145(4):514-23.

[2] Liu Y, Ma J, Yu K, Li M, Liu F, Yan Q, Wang Z, Guo S. Expression of programmed cell death 1/programmed cell death ligand 1 in the tumor microenvironments of primary gastrointestinal diffuse large B cell lymphomas. Pathol Res Pract. 2018;214(4):507-512.

[3] Chen Z, Deng X, Ye Y, Gao L, Zhang W, Liu W, Zhao S. Novel risk stratification of de novo diffuse large B cell lymphoma based on tumour-infiltrating T lymphocytes evaluated by flow cytometry. Ann Hematol 2019; 98:391-399.
